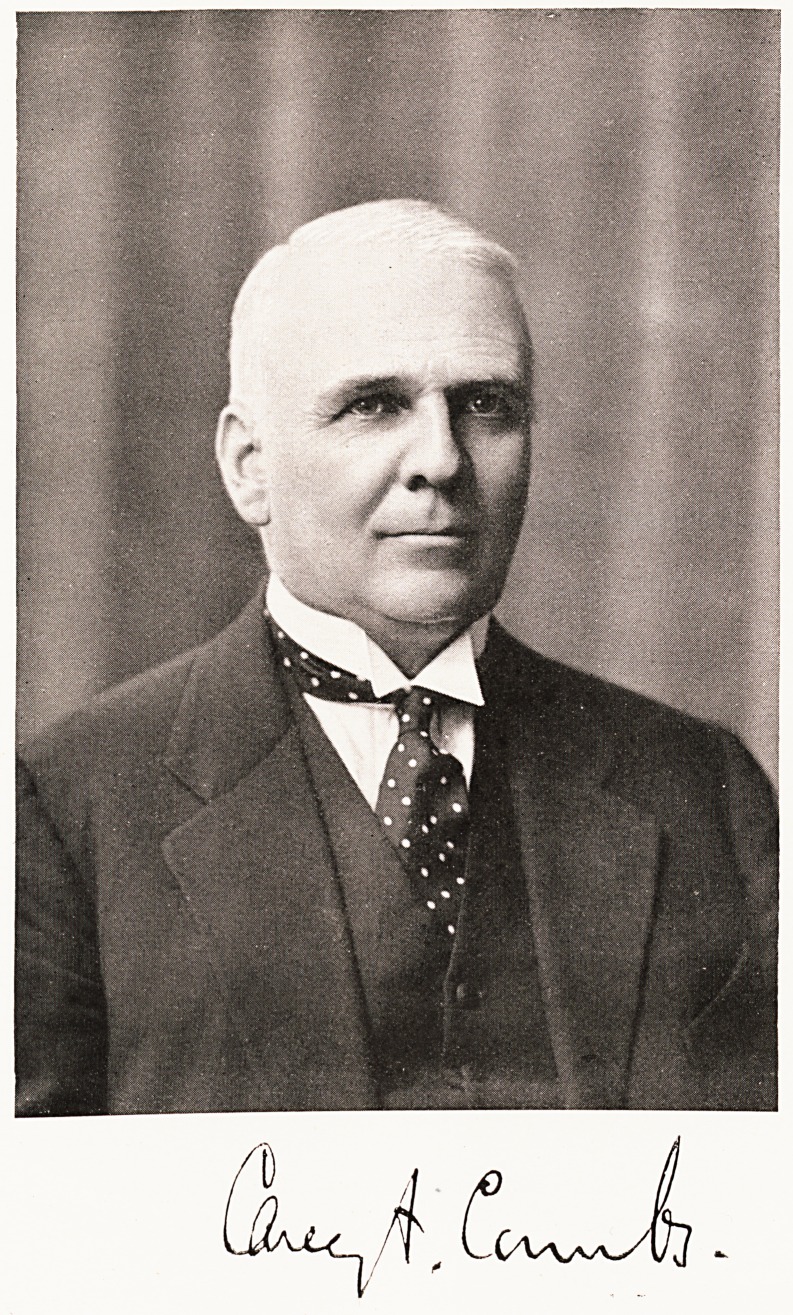# Dr. Carey Franklin Coombs

**Published:** 1932

**Authors:** 


### Obituary

### DR. CAREY FRANKLIN COOMBS.

WE have to record with the deepest regret the death of Dr. Carey Coombs. He passed away in the General Hospital on 9th December, after an illness of some five weeks' duration. It will be remembered that he was taken ill suddenly in the street at the beginning of November. Though he appeared to make favourable progress, it was realized by those in attendance that his condition was very grave and that the chances of recovery were remote. The end came suddenly and as a great shock to his many friends.

With the passing of Carey Coombs Bristol has lost a great man and the world of medicine a great physician and teacher. Our first feeling is a sense of irreparable loss ; but surely we may find inspiration and encouragement in the reflection that Coombs lived—yes, lived every moment of his life—and

Carey A. Combs.

has left us a lasting memorial. His work for medical science lives on and will endure. His contributions to medical literature will rank among the classics of cardiology. His book, *Rheumatic Heart Disease*, and his Lumleian Lecture on "Cardiovascular Syphilis" are contributions to medical knowledge which will not readily be superseded. At the time of his death he was engaged in an extensive research on "Heart Disease." Under the auspices of the Harmsworth Memorial Trust he was conducting an inquiry into the ætiology and prevention of ulcerative endocarditis. Coombs quickly realized that the problems presented by ulcerative endocarditis itself were well-nigh hopeless, and that prevention could only be achieved by tackling the disease at its source, that is to say, by preventing simple endocarditis. He had collected a wealth of material which was due to appear shortly in book form. It is hoped that the work may yet be carried through to completion.

Carey Coombs lived at high pressure always. How he got through the amount of work he did was a source of amazement and no little anxiety to his colleagues. Although he conducted a large and successful consulting practice, it may truthfully be said that his heart was in his hospital and scientific work. In later years high academic distinctions poured in upon him. A detailed account of his career is one record of achievement. He was born in 1879 at Castle Cary, Somerset, the son of Dr. Carey Pearce Coombs, of Frome. As a student he was educated at the Bristol University College and St. Mary's Hospital, London. At the latter he gained the General Proficiency Scholarship and Cheadle Medal. He qualified with the M.B., B.S. in 1901, taking honours in Medicine and Obstetrics; he took the M.D. 1903, M.R.C.P. 1908, and F.R.C.P. 1917. At St. Mary's he held numerous house appointments, including that of Medical Registrar. Later he came to Bristol as Registrar to the Children's Hospital. In 1907 he became Assistant Physician at the Bristol General Hospital. In 1920 he became full physician. In recent years he filled many other roles. In 1926 he became

Assistant Editor of the *Bristol Medico-Chirurgical Journal,* 1925 he delivered the Long Fox Lecture, 1927 the Chadwick Lecture, and the Lumleian Lecture in 1930. At the time of his death he was a Councillor of the Royal College of Physicians. In 1930 he was elected President of the Bath and Bristol Branch of the British Medical Association.

During the war Coombs had a long record of faithful service in Mesopotamia, in France, and in England towards the end of the war. In Mesopotamia he contracted a serious illness, which may have been a factor determining his more recent breakdown and premature death.

As a colleague and friend Coombs will be greatly missed by all who knew him, and he will ever be remembered with the sincerest affection. He was an incurable optimist, full of vitality and energy. He appeared to have an insatiable capacity for formulating schemes for the organization of research and medical services of the City. He was one of the chief promoters of the New Hospital at Winford, and founded a large cardiac clinic in association with the Education Committee.

During the last few weeks of his life a great part of his time was spent in making plans for the future. He realized how greatly his activities would have to be restricted, but it was his hospital and research work which he placed in the foreground, and he was always intolerant of suggestions which involved any curtailment of these activities.

It is certain that no one can fill the gap that is left by the loss of Carey Coombs, but the best tribute that we can pay to his memory is to see that some at least of the work in which he was so deeply interested is carried on.

## Figures and Tables

**Figure f1:**